# 2,3-Dichloro-5,8-dimeth­oxy-1,4-naphtho­quinone

**DOI:** 10.1107/S1600536812023926

**Published:** 2012-06-13

**Authors:** Maraizu Ukaegbu, Ray J. Butcher, N. M. Enwerem, Oladapo Bakare, Charles Hosten

**Affiliations:** a Department of Chemistry, Howard University, 525 College Street, NW, Washington, DC, 2059 USA

## Abstract

In the crystal structure of the title compound, C_12_H_8_Cl_2_O_4_, mol­ecules crystallize in planes parallel to (-204) with an inter­planar distance of 3.288 (2) Å [centroid–centroid distance = 3.819 (2) and slippage = 1.932 (2) Å]. The structure features C—H⋯O inter­actions involving meth­oxy and aromatic H atoms and the carbonyl O atoms as well as a C—H⋯Cl inter­action involving an aromatic H atom. In addition there are short inter­halogen contacts between adjoining mol­ecules [Cl⋯Cl = 3.3709 (5) Å].

## Related literature

For biological properties of the title compound, see: Huang *et al.* (1998 ▶); Copeland *et al.* (2007 ▶); Lien *et al.* (1997 ▶). For structures of related 2,3-dichloro-1,4-naphtho­quinone derivatives, see: Ikemoto *et al.* (1977 ▶); Rubio *et al.* (1985 ▶). For quinoid systems, see: Driebergen *et al.* (1986 ▶); Scheuermann *et al.* (2009 ▶).
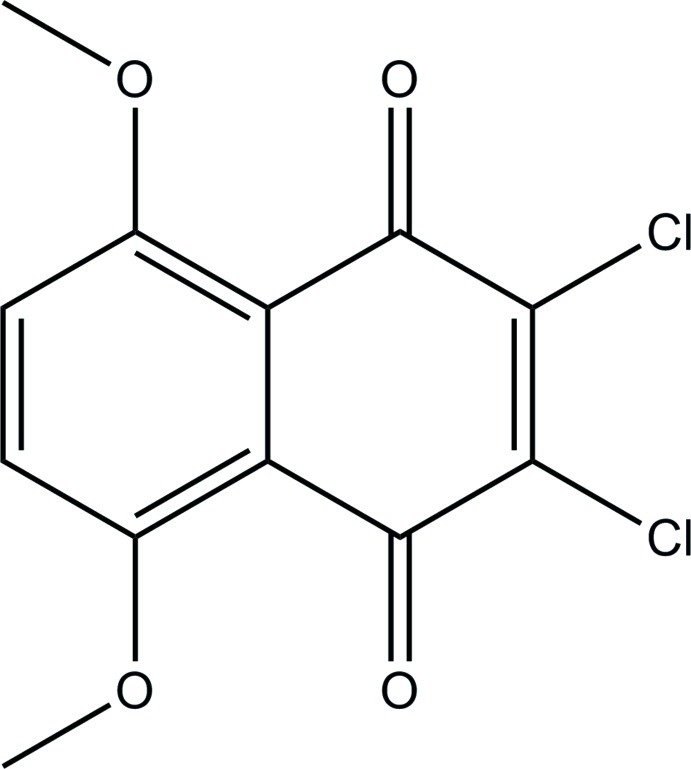


## Experimental

### 

#### Crystal data

C_12_H_8_Cl_2_O_4_
*M* *_r_* = 287.08
Monoclinic, 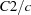

*a* = 9.9366 (2) Å
*b* = 15.6564 (3) Å
*c* = 14.8505 (3) Å
β = 103.782 (2)°
*V* = 2243.79 (8) Å^3^
*Z* = 8
Cu *K*α radiation
μ = 5.27 mm^−1^
*T* = 123 K
0.81 × 0.30 × 0.23 mm

#### Data collection

Oxford Diffraction Xcalibur Ruby Gemini diffractometer
Absorption correction: analytical (*CrysAlis PRO*; Oxford Diffraction, 2007 ▶) *T*_min_ = 0.154, *T*_max_ = 0.418
8037 measured reflections
2199 independent reflections
2156 reflections with *I* > 2σ(*I*)
*R*_int_ = 0.024

#### Refinement

*R*[*F* ^2^ > 2σ(*F* ^2^)] = 0.028
*wR*(*F* ^2^) = 0.077
*S* = 1.09
2199 reflections
166 parameters
H-atom parameters constrained
Δρ_max_ = 0.31 e Å^−3^
Δρ_min_ = −0.27 e Å^−3^

### 

Data collection: *CrysAlis PRO* (Oxford Diffraction, 2007 ▶); cell refinement: *CrysAlis PRO*; data reduction: *CrysAlis PRO*; program(s) used to solve structure: *SHELXS97* (Sheldrick, 2008 ▶); program(s) used to refine structure: *SHELXL97* (Sheldrick, 2008 ▶); molecular graphics: *SHELXTL* (Sheldrick, 2008 ▶); software used to prepare material for publication: *SHELXTL*.

## Supplementary Material

Crystal structure: contains datablock(s) I, New_Global_Publ_Block. DOI: 10.1107/S1600536812023926/bt5932sup1.cif

Structure factors: contains datablock(s) I. DOI: 10.1107/S1600536812023926/bt5932Isup2.hkl

Additional supplementary materials:  crystallographic information; 3D view; checkCIF report

## Figures and Tables

**Table 1:** Hydrogen-bond geometry (Å, °)

*D*—H⋯*A*	*D*—H	H⋯*A*	*D*⋯*A*	*D*—H⋯*A*
C7—H7*A*⋯Cl2^i^	0.95	2.72	3.6593 (14)	169
C8—H8*A*⋯O2^i^	0.95	2.54	3.3337 (18)	142
C11—H11*C*⋯O1^ii^	0.98	2.62	3.4030 (19)	137
C12—H12*A*⋯O1^iii^	0.98	2.49	3.3723 (19)	149

Symmetry codes: (i) 
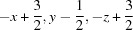
; (ii) 
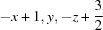
; (iii) 
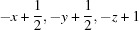
.

**Fig. 1.**
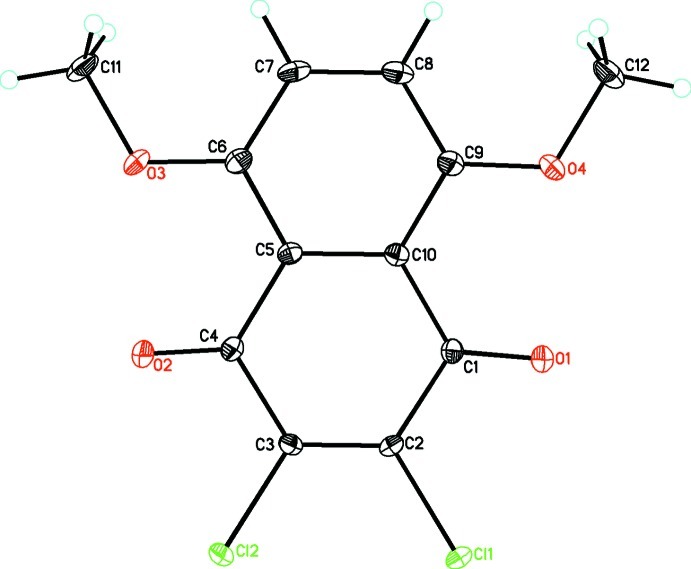
Diagram of C12H8Cl2O4 with atomic displacement parameters drawn at 30% probability.

**Fig. 2.**
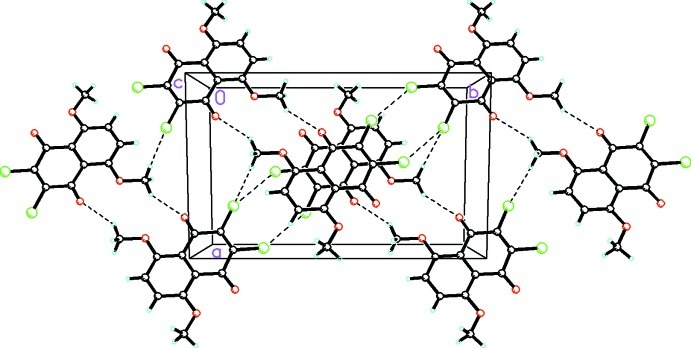
The molecular packing for C12H8Cl2O4 viewed along the c axis.

## supplementary crystallographic information

### Comment

Compounds with the methoxy and chloro groups on the 1, 4-naphthoquinone skeleton were reported to show inhibitory effects on cancerous cells (Huang *et al.*, 1998; Lien *et al.*, 1997). 2,3-Dichloro-5,8-dimethoxy-1,4-naphthoquinone, C_12_H_8_Cl_2_O_4_, was synthesized as a potential anticancer agent and has been reported to exhibit anti-inflammatory, antiplatelet, anti-allergic and anticancer activities (Huang *et al.*, 1998; Copeland *et al.*, 2007). This biological function is based on chemical properties inherent in molecule. To understand its biological behavior, therefore, it is of great importance to determine the structural property and its molecular dimensions. The methoxy and chloro group in the quinoid ring give it interesting redox and biological properties as well as an excellent coordination site (Driebergen *et al.*, 1986; Scheuermann *et al.*, 2009). The coordinating potential of the molecule could be used as a tool for the formation of new organometallic compounds (Scheuermann *et al.*, 2009).

The molecules in the title compound crystallize in planes parallel to (-2 0 4) with an interplanar distance of 3.288Å forming a charge transfer complex. The distance between the overlapping planes of neighboring molecules is 3.385 (3) Å and 3.653 (3) Å. There are intermolecular interactions between both a methoxy hydrogen and an aromatic hydrogen and the carbonyl O atoms. Intermolecular interactions are also observed between chlorine atom and the aromatic and methoxy H atoms. In addition there are short interhalogen contacts between adjoining molecules (Cl1···Cl2 3.3709 (5) Å) These C—H···Cl, C—H···O and Cl···Cl interactions in the crystal structure link the molecules to produce a three dimensional network.

### Experimental

2,3-Dichloro-5,8-dimethoxy-1,4-naphthoquinone (DDNQ) was synthesized as described by Huang (Huang *et al.*, 1998). The reaction of dichloromaleic anhydride and 1,4-dimethoxybenzene produces a mixture of 6,7-dichloro-5,8-dihydroxy-1,4-naphthoquinone and 2,3-dichloro-5,8-dihydroxy-1,4-naphthoquinone. *o*-Methylation of 6,7-dichloro-5,8-dihydroxy-1,4-naphthoquinone and 2,3-dichloro-5,8-dihydroxy-1,4-naphthoquinone with methyl iodide-silver oxide produces the mixture of 6,7-dichloro-5,8-dimethoxy-1,4-naphthoquinone (1) and 2,3-dichloro-5,8-dimethoxy-1,4-naphthoquinone (2). The mixture of 1 and 2 were separated by column chromatography on silica gel to obtain pure 2,3-dichloro-5,8-dimethoxy-1,4-naphthoquinone (DDNQ). Solid DDNQ was recrystallized in dichloromethane to produce X-ray diffraction quality crystals.

### Refinement

H atoms were placed in geometrically idealized positions and constrained to ride on their parent atoms with a C—H distance of 0.95 Å *U*_iso_(H) = 1.2*U*_eq_(C) and 0.98 Å for CH_3_ [*U*_iso_(H) = 1.5*U*_eq_(C)].

### Crystal data

**Table:**

C_12_H_8_Cl_2_O_4_	*F*(000) = 1168
*M**_r_* = 287.08	*D*_x_ = 1.700 Mg m^−^^3^
Monoclinic, *C*2/*c*	Cu *K*α radiation, λ = 1.54184 Å
Hall symbol: -C 2yc	Cell parameters from 6065 reflections
*a* = 9.9366 (2) Å	θ = 3.1–72.4°
*b* = 15.6564 (3) Å	µ = 5.27 mm^−^^1^
*c* = 14.8505 (3) Å	*T* = 123 K
β = 103.782 (2)°	Slab, pink
*V* = 2243.79 (8) Å^3^	0.81 × 0.30 × 0.23 mm
*Z* = 8	

### Data collection

**Table:**

Oxford Diffraction Xcalibur Ruby Gemini diffractometer	2199 independent reflections
Radiation source: Enhance (Cu) X-ray Source	2156 reflections with *I* > 2σ(*I*)
Graphite monochromator	*R*_int_ = 0.024
Detector resolution: 10.5081 pixels mm^-1^	θ_max_ = 72.5°, θ_min_ = 5.4°
ω scans	*h* = −12→12
Absorption correction: analytical (*CrysAlis PRO*; Oxford Diffraction, 2007)	*k* = −19→18
*T*_min_ = 0.154, *T*_max_ = 0.418	*l* = −13→18
8037 measured reflections	

### Refinement

**Table:**

Refinement on *F*^2^	Secondary atom site location: difference Fourier map
Least-squares matrix: full	Hydrogen site location: inferred from neighbouring sites
*R*[*F*^2^ > 2σ(*F*^2^)] = 0.028	H-atom parameters constrained
*wR*(*F*^2^) = 0.077	*w* = 1/[σ^2^(*F*_o_^2^) + (0.0452*P*)^2^ + 1.7012*P*] where *P* = (*F*_o_^2^ + 2*F*_c_^2^)/3
*S* = 1.09	(Δ/σ)_max_ = 0.001
2199 reflections	Δρ_max_ = 0.31 e Å^−^^3^
166 parameters	Δρ_min_ = −0.27 e Å^−^^3^
0 restraints	Extinction correction: *SHELXL*, Fc^*^=kFc[1+0.001xFc^2^λ^3^/sin(2θ)]^-1/4^
Primary atom site location: structure-invariant direct methods	Extinction coefficient: 0.00077 (11)

### Special details

**Table:**

Geometry. All e.s.d.'s (except the e.s.d. in the dihedral angle between two l.s. planes) are estimated using the full covariance matrix. The cell e.s.d.'s are taken into account individually in the estimation of e.s.d.'s in distances, angles and torsion angles; correlations between e.s.d.'s in cell parameters are only used when they are defined by crystal symmetry. An approximate (isotropic) treatment of cell e.s.d.'s is used for estimating e.s.d.'s involving l.s. planes.
Refinement. Refinement of *F*^2^ against ALL reflections. The weighted *R*-factor *wR* and goodness of fit *S* are based on *F*^2^, conventional *R*-factors *R* are based on *F*, with *F* set to zero for negative *F*^2^. The threshold expression of *F*^2^ > σ(*F*^2^) is used only for calculating *R*-factors(gt) *etc*. and is not relevant to the choice of reflections for refinement. *R*-factors based on *F*^2^ are statistically about twice as large as those based on *F*, and *R*- factors based on ALL data will be even larger.

### Fractional atomic coordinates and isotropic or equivalent isotropic displacement parameters (Å^2^)

**Table:**

	*x*	*y*	*z*	*U*_iso_*/*U*_eq_	
Cl1	0.22986 (3)	0.61933 (2)	0.50894 (2)	0.02353 (13)	
Cl2	0.47240 (4)	0.73207 (2)	0.62182 (2)	0.02670 (14)	
O1	0.29765 (11)	0.44185 (6)	0.51438 (7)	0.0247 (2)	
O2	0.70729 (11)	0.63328 (7)	0.71429 (7)	0.0256 (2)	
O3	0.83562 (11)	0.50132 (7)	0.79392 (7)	0.0266 (3)	
O4	0.41728 (11)	0.30361 (6)	0.58176 (7)	0.0246 (2)	
C1	0.39013 (13)	0.48023 (9)	0.56736 (9)	0.0163 (3)	
C2	0.37962 (13)	0.57504 (9)	0.57239 (9)	0.0163 (3)	
C3	0.48343 (14)	0.62303 (8)	0.62041 (9)	0.0169 (3)	
C4	0.61546 (13)	0.58551 (9)	0.67577 (9)	0.0166 (3)	
C5	0.62262 (13)	0.49062 (9)	0.68126 (9)	0.0160 (3)	
C6	0.73632 (14)	0.45098 (9)	0.74118 (9)	0.0194 (3)	
C7	0.74226 (15)	0.36157 (10)	0.74499 (10)	0.0233 (3)	
H7A	0.8192	0.3346	0.7852	0.028*	
C8	0.63901 (16)	0.31218 (9)	0.69163 (10)	0.0232 (3)	
H8A	0.6467	0.2517	0.6946	0.028*	
C9	0.52280 (15)	0.34971 (9)	0.63305 (10)	0.0193 (3)	
C10	0.51411 (14)	0.43964 (9)	0.62756 (9)	0.0162 (3)	
C11	0.94626 (15)	0.46147 (11)	0.86031 (11)	0.0291 (3)	
H11A	1.0087	0.5054	0.8939	0.044*	
H11B	0.9978	0.4235	0.8282	0.044*	
H11C	0.9079	0.4282	0.9042	0.044*	
C12	0.42089 (18)	0.21201 (9)	0.59175 (12)	0.0295 (3)	
H12A	0.3370	0.1873	0.5518	0.044*	
H12B	0.4255	0.1970	0.6565	0.044*	
H12C	0.5027	0.1894	0.5737	0.044*	

### Atomic displacement parameters (Å^2^)

**Table:**

	*U*^11^	*U*^22^	*U*^33^	*U*^12^	*U*^13^	*U*^23^
Cl1	0.01650 (19)	0.0218 (2)	0.0275 (2)	0.00482 (11)	−0.00425 (14)	0.00124 (12)
Cl2	0.0297 (2)	0.01223 (19)	0.0314 (2)	0.00149 (12)	−0.00606 (15)	−0.00074 (12)
O1	0.0218 (5)	0.0195 (5)	0.0281 (5)	−0.0027 (4)	−0.0033 (4)	−0.0024 (4)
O2	0.0204 (5)	0.0211 (5)	0.0300 (6)	−0.0041 (4)	−0.0045 (4)	−0.0011 (4)
O3	0.0190 (5)	0.0279 (6)	0.0263 (5)	0.0043 (4)	−0.0075 (4)	0.0011 (4)
O4	0.0260 (5)	0.0131 (5)	0.0331 (6)	−0.0016 (4)	0.0036 (4)	−0.0014 (4)
C1	0.0152 (6)	0.0171 (7)	0.0167 (6)	−0.0015 (5)	0.0041 (5)	−0.0003 (5)
C2	0.0131 (6)	0.0182 (7)	0.0163 (6)	0.0032 (5)	0.0009 (5)	0.0019 (5)
C3	0.0194 (7)	0.0124 (7)	0.0182 (7)	0.0012 (5)	0.0030 (5)	0.0004 (5)
C4	0.0156 (6)	0.0192 (7)	0.0146 (6)	−0.0003 (5)	0.0024 (5)	0.0004 (5)
C5	0.0146 (6)	0.0173 (7)	0.0164 (6)	0.0024 (5)	0.0041 (5)	0.0015 (5)
C6	0.0165 (6)	0.0238 (7)	0.0181 (7)	0.0029 (5)	0.0042 (5)	0.0014 (5)
C7	0.0196 (7)	0.0248 (7)	0.0256 (7)	0.0093 (6)	0.0055 (6)	0.0090 (6)
C8	0.0244 (7)	0.0172 (7)	0.0298 (8)	0.0050 (5)	0.0104 (6)	0.0059 (6)
C9	0.0201 (7)	0.0177 (7)	0.0214 (7)	0.0006 (5)	0.0076 (5)	0.0009 (5)
C10	0.0168 (6)	0.0155 (7)	0.0172 (6)	0.0011 (5)	0.0057 (5)	0.0010 (5)
C11	0.0202 (7)	0.0375 (9)	0.0244 (7)	0.0104 (6)	−0.0050 (6)	0.0029 (6)
C12	0.0359 (9)	0.0140 (7)	0.0406 (9)	−0.0018 (6)	0.0129 (7)	−0.0020 (6)

### Geometric parameters (Å, º)

**Table:**

Cl1—C2	1.7074 (13)	C5—C10	1.4233 (19)
Cl2—C3	1.7111 (13)	C6—C7	1.402 (2)
O1—C1	1.2166 (17)	C7—C8	1.375 (2)
O2—C4	1.2126 (17)	C7—H7A	0.9500
O3—C6	1.3564 (18)	C8—C9	1.399 (2)
O3—C11	1.4331 (17)	C8—H8A	0.9500
O4—C9	1.3492 (18)	C9—C10	1.4117 (18)
O4—C12	1.4413 (17)	C11—H11A	0.9800
C1—C10	1.4819 (19)	C11—H11B	0.9800
C1—C2	1.4911 (18)	C11—H11C	0.9800
C2—C3	1.3367 (19)	C12—H12A	0.9800
C3—C4	1.4927 (18)	C12—H12B	0.9800
C4—C5	1.4886 (18)	C12—H12C	0.9800
C5—C6	1.4052 (19)		
			
C6—O3—C11	118.53 (12)	C6—C7—H7A	119.3
C9—O4—C12	118.51 (12)	C7—C8—C9	120.94 (13)
O1—C1—C10	124.72 (13)	C7—C8—H8A	119.5
O1—C1—C2	118.22 (12)	C9—C8—H8A	119.5
C10—C1—C2	117.05 (11)	O4—C9—C8	122.80 (13)
C3—C2—C1	122.14 (12)	O4—C9—C10	118.19 (12)
C3—C2—Cl1	121.77 (11)	C8—C9—C10	119.00 (13)
C1—C2—Cl1	116.03 (10)	C9—C10—C5	119.95 (12)
C2—C3—C4	122.54 (12)	C9—C10—C1	119.55 (12)
C2—C3—Cl2	121.59 (11)	C5—C10—C1	120.49 (12)
C4—C3—Cl2	115.87 (10)	O3—C11—H11A	109.5
O2—C4—C5	124.68 (12)	O3—C11—H11B	109.5
O2—C4—C3	118.74 (12)	H11A—C11—H11B	109.5
C5—C4—C3	116.56 (11)	O3—C11—H11C	109.5
C6—C5—C10	119.64 (13)	H11A—C11—H11C	109.5
C6—C5—C4	119.75 (12)	H11B—C11—H11C	109.5
C10—C5—C4	120.60 (12)	O4—C12—H12A	109.5
O3—C6—C7	122.63 (13)	O4—C12—H12B	109.5
O3—C6—C5	118.27 (13)	H12A—C12—H12B	109.5
C7—C6—C5	119.10 (13)	O4—C12—H12C	109.5
C8—C7—C6	121.33 (13)	H12A—C12—H12C	109.5
C8—C7—H7A	119.3	H12B—C12—H12C	109.5
			
O1—C1—C2—C3	−172.16 (13)	C4—C5—C6—C7	−179.42 (12)
C10—C1—C2—C3	7.53 (19)	O3—C6—C7—C8	179.06 (13)
O1—C1—C2—Cl1	5.05 (16)	C5—C6—C7—C8	−0.2 (2)
C10—C1—C2—Cl1	−175.27 (9)	C6—C7—C8—C9	−1.4 (2)
C1—C2—C3—C4	−2.5 (2)	C12—O4—C9—C8	3.6 (2)
Cl1—C2—C3—C4	−179.59 (10)	C12—O4—C9—C10	−175.82 (12)
C1—C2—C3—Cl2	177.39 (10)	C7—C8—C9—O4	−177.91 (13)
Cl1—C2—C3—Cl2	0.34 (17)	C7—C8—C9—C10	1.5 (2)
C2—C3—C4—O2	176.86 (13)	O4—C9—C10—C5	179.39 (12)
Cl2—C3—C4—O2	−3.09 (17)	C8—C9—C10—C5	−0.06 (19)
C2—C3—C4—C5	−4.63 (19)	O4—C9—C10—C1	0.79 (18)
Cl2—C3—C4—C5	175.42 (9)	C8—C9—C10—C1	−178.66 (12)
O2—C4—C5—C6	6.3 (2)	C6—C5—C10—C9	−1.52 (19)
C3—C4—C5—C6	−172.15 (12)	C4—C5—C10—C9	179.58 (11)
O2—C4—C5—C10	−174.84 (13)	C6—C5—C10—C1	177.06 (11)
C3—C4—C5—C10	6.75 (18)	C4—C5—C10—C1	−1.83 (18)
C11—O3—C6—C7	−4.1 (2)	O1—C1—C10—C9	−6.9 (2)
C11—O3—C6—C5	175.23 (12)	C2—C1—C10—C9	173.40 (11)
C10—C5—C6—O3	−177.66 (12)	O1—C1—C10—C5	174.48 (12)
C4—C5—C6—O3	1.24 (18)	C2—C1—C10—C5	−5.19 (18)
C10—C5—C6—C7	1.67 (19)		

### Hydrogen-bond geometry (Å, º)

**Table:**

*D*—H···*A*	*D*—H	H···*A*	*D*···*A*	*D*—H···*A*
C7—H7*A*···Cl2^i^	0.95	2.72	3.6593 (14)	169
C8—H8*A*···O2^i^	0.95	2.54	3.3337 (18)	142
C11—H11*C*···O1^ii^	0.98	2.62	3.4030 (19)	137
C12—H12*A*···O1^iii^	0.98	2.49	3.3723 (19)	149

Symmetry codes: (i) −*x*+3/2, *y*−1/2, −*z*+3/2; (ii) −*x*+1, *y*, −*z*+3/2; (iii) −*x*+1/2, −*y*+1/2, −*z*+1.
